# Socio-Demographic Variables, Fear of COVID-19, Anxiety, and Depression: Prevalence, Relationships and Explanatory Model in the General Population of Seven Latin American Countries

**DOI:** 10.3389/fpsyg.2021.695989

**Published:** 2021-11-05

**Authors:** Tomás Caycho-Rodríguez, José M. Tomás, Lindsey W. Vilca, Carlos Carbajal-León, Mauricio Cervigni, Miguel Gallegos, Pablo Martino, Ignacio Barés, Manuel Calandra, César Armando Rey Anacona, Claudio López-Calle, Rodrigo Moreta-Herrera, Edgardo René Chacón-Andrade, Marlon Elías Lobos-Rivera, Perla del Carpio, Yazmín Quintero, Erika Robles, Macerlo Panza Lombardo, Olivia Gamarra Recalde, Andrés Buschiazzo Figares, Michael White, Carmen Burgos Videla

**Affiliations:** ^1^Facultad de Ciencias de la Salud, Universidad Privada del Norte, Trujillo, Peru; ^2^Department of Methodology for the Behavioral Sciences, University of Valencia, Valencia, Spain; ^3^Departamento de Psicología, Peruvian Union University, Lima, Peru; ^4^Facultad de Psicología, National University of Rosario, Rosario, Argentina; ^5^Centro Interdisciplinario de Investigaciones en Ciencias de la Salud y del Comportamiento, Consejo Nacional de Investigaciones Científicas y Técnicas, Buenos Aires, Argentina; ^6^Facultad de Ciencias de la Salud, Catholic University of the Maule, Maule, Chile; ^7^Consejo Nacional de Investigaciones Científicas y Técnicas, Buenos Aires, Argentina; ^8^School of Psychology, Pedagogical and Technological University of Colombia, Tunja, Colombia; ^9^Facultad de Psicología, University of Cuenca, Cuenca, Ecuador; ^10^Escuela de Psicología, Pontificia Universidad Católica del Ecuador, Quito, Ecuador; ^11^Escuela de Psicología, Facultad de Ciencias Sociales, Universidad Tecnológica de El Salvador, San Salvador, El Salvador; ^12^Department of Social Studies, University of Guanajuato, Guanajuato, Mexico; ^13^Faculty of Behavioral Sciences, University Autonomous of the State of Mexico, Toluca, Mexico; ^14^Faculty of Health Sciences, Universidad Nacional del Este, Ciudad del Este, Paraguay; ^15^Sensorium, Ciudad del Este, Paraguay; ^16^Centro de Estudios Adlerianos, Montevideo, Uruguay; ^17^Dirección General de Investigación, Peruvian Union University, Lima, Peru; ^18^Instituto de Investigación en Ciencias Sociales y Educación, Universidad de Atacama, Copiapó, Chile

**Keywords:** anxiety, depression, fear of COVID-19, Latin America, socio-demographic

## Abstract

The COVID-19 pandemic has gravely impacted Latin America. A model was tested that evaluated the contribution of socio-demographic factors and fear of COVID-19 on anxiety and depression in samples of residents in seven Latin American countries (Argentina, Ecuador, Mexico, Paraguay, Uruguay, Colombia, and El Salvador). A total of 4,881 individuals, selected by convenience sampling, participated in the study. Moderate and severe levels of depressive symptoms and anxiety were identified, as well as a moderate average level of fear of COVID-19. In addition, it was observed that about a quarter of the participants presented symptoms of generalized anxiety disorder and a major depressive episode. Fear of COVID-19 significantly and positively predicted anxiety and depressive symptoms, whereas the effects of socio-demographic variables are generally low [χ^2^(287) = 5936.96, *p* < 0.001; RMSEA = 0.064 [0.062, 0.065]; CFI = 0.947; and SRMR = 0.050]. This suggests the need for the implementation of preventive actions in the general population of these countries, with the aim of reducing the prevalence of depressive, anxious and fearful symptoms related to COVID-19.

## Introduction

Latin America and the Caribbean (LAC) includes 33 countries, mostly low and middle-income, with a population of over 658 million inhabitants, representing 8.6% of the total world population and expected to reach 721 million inhabitants by 2030 (Errazuriz and Crisostomo, 2021). Since its appearance at the end of 2019, COVID-19 spread from China to the rest of the countries in the world, with Latin America and the Caribbean (LAC) being the last region to have cases diagnosed with the disease (Pablos-Méndez et al., 2020). Specifically, on February 25, 2020, the first case of COVID-19 in LAC was confirmed in Brazil (Rodriguez-Morales et al., 2020). A few weeks later, most LAC countries took measures to prevent the spread of the disease in their territory, such as border closures, mandatory social isolation, curfews, and cancelation of intraprovincial travel (Burki, 2020; Miller et al., 2020). Even so, the number of diagnosed cases in the region continued to increase. According to the Coronavirus Resource Center at Johns Hopkins University, as of February 22, 2021, a total of 20,747,458 cases of COVID-19 were reported in LAC, with Brazil being the country most affected by this pandemic in the region, with about 10.2 million confirmed cases, followed by Colombia with more than 2.2 million infected and Mexico with a total of 2.04 million cases. Other Latin American countries heavily affected by COVID-19 are Argentina, Peru, Chile and Ecuador. Likewise, the majority of COVID-19 deaths recorded in LAC occurred in Brazil (246,504 deaths) and Mexico (180,107 deaths) (Coronavirus Resource Center, 2020). This has made LAC one of the most severely affected regions by the COVID-19 pandemic (Gallegos et al., 2020; Garcia et al., 2020).

The limited economic resources and deficient health services make the situation of the population in several LAC countries particularly alarming, generating difficulties in identifying possible cases of COVID-19, mitigating its spread and providing adequate treatment to patients (Rodríguez-Hidalgo et al., 2020). This has generated a context of great socio-health vulnerability, which can especially affect the mental health of the population (Llibre-Guerra et al., 2020). Internationally, several studies have reported that the increase in the number of cases and deaths due to COVID-19, together with actions such as social distancing and isolation, have generated a higher prevalence of depression, anxiety, post-traumatic stress disorder, fear and insomnia during the COVID outbreak, especially in contexts of social and economic vulnerability (da Silva et al., 2020; Hossain et al., 2020; Kontoangelos et al., 2020; Rajkumar, 2020; Vindegaard and Benros, 2020; Xiong et al., 2020). In LAC, Brazil reported an 81.90% prevalence of anxiety, 68% for depression, 64.50% for anger, somatic symptoms at 62.60% and sleep disturbances at 55.30% (Goularte et al., 2021). In Colombia, 14.3% of the adult population expressed high perceived stress (Pedrozo-Pupo et al., 2020); while in Peru, a prevalence of 30.80% of depressive symptoms, 41.80% of anxiety and 34.10% of stress was observed (Concha et al., 2020). Likewise, in El Salvador, about 75% of people over 18 years of age reported having mild symptoms of depression, anxiety and stress; while a quarter experienced moderate and severe emotional symptoms during the social isolation period (Orellana and Orellana, 2020). Finally, in Cuba, it was found that 30.96 and 26.90% of the participants had high and medium levels of anxiety, respectively; 36.54% and 13.70% manifested medium and high levels of depression, respectively; while 66.49% presented altered stress levels (Arias Molina et al., 2020).

A characteristic emotion of pandemic-type viral infections, and one that is associated with alterations in mental health, is the fear that can be generated in a large part of the population (Ahorsu et al., 2020). Fear is a basic and fundamental emotion for survival, which is presented as a response to a specific and imminent perceived threat (Schimmenti et al., 2020; Starcevic et al., 2020). Studies indicate that feeling at risk of being infected allows for greater engagement in certain health prevention behaviors, such as hand washing and maintaining social distancing during the early stages of a pandemic (Wise et al., 2020). Inversely, the absence of fear can be detrimental, generating a decrease in hygiene behaviors and leading to ignoring measures aimed at mitigating the spread of the disease (Taylor, 2019). On the other hand, when fear is excessive it could become maladaptive (Mertens et al., 2020), having the potential to generate phobias, as well as higher levels of depression, anxiety, stress and addictive substance use (Asmundson and Taylor, 2020; Bitan et al., 2020; Caycho-Rodríguez et al., 2020, 2021b; Doshi et al., 2020; Haktanir et al., 2020; Sakib et al., 2020). The scientific literature points out that fear of COVID-19 is related to a greater extent to anxiety and to a lesser extent to depression (Ahorsu et al., 2020; Bitan et al., 2020). A recent study that evaluated fear of COVID-19 in seven Latin American countries (Argentina, Ecuador, Colombia, Mexico, El Salvador, Uruguay, and Paraguay), reported that the emotional and physiological reactions to fear differed significantly between countries, where the differences were small between Colombia, Ecuador, El Salvador, Mexico, and Paraguay; but in Argentina and Uruguay fear was much lower than the other countries (Caycho-Rodríguez et al., 2021b).

Likewise, in the current health crisis, evidence has suggested the importance of some socio-demographic variables as predictors of mental health. For example, women and younger people reported higher levels of anxiety, depression and fear during the COVID-19 pandemic (Andrade et al., 2020; Bäuerle et al., 2020; Broche-Pérez et al., 2020; Elbay et al., 2020; Haktanir et al., 2020; Vindegaard and Benros, 2020; Caycho-Rodríguez et al., 2021a). However, other studies report contrary findings, reporting no differences in fear of COVID-19 based on age (Soraci et al., 2020) or reporting higher levels of fear of becoming infected with COVID-19 in older compared to younger people (de Leo and Trabucchi, 2020; Meng et al., 2020). On the other hand, people who were single, separated, divorced and/or widowed were more likely to have higher mental health frailty (Smith et al., 2020; Ustun, 2020). However, it has also been reported that there are no statistically significant differences in depression and anxiety in individuals with different marital statuses (Wu et al., 2020). In fact, some studies even suggest that marital status positively predicts fear of COVID-19 (Mohammadpour et al., 2020) and that being married increases disease-related fear (Doshi et al., 2020).

**FIGURE 1:**
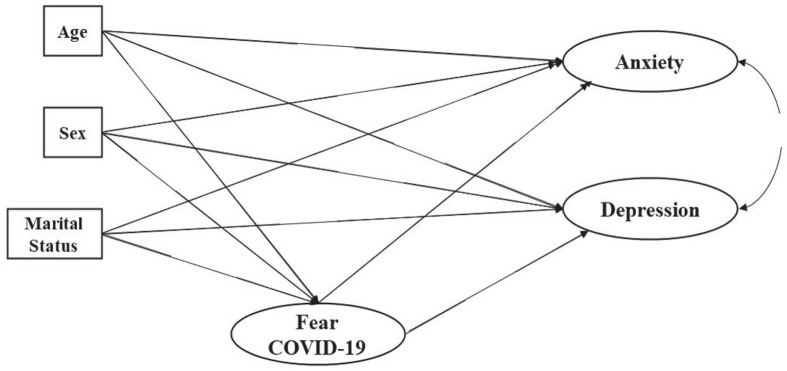
Models of fear of COVID-19 predicting anxiety and depression. A double headed arrow indicates a covariance, whereas single headed arrows indicate a hypothetical predictive effect between two variables.

Given that the COVID-19 pandemic is a global problem affecting different countries, a cross-national understanding of possible socio-demographic and emotional predictors of anxiety and depression is imperative. Therefore, the primary objective of the present study was to test a structural equation model that assesses the contribution of socio-demographic factors (sex, age, and marital status) and fear of COVID-19 on anxiety and depression, as well as to study their potential invariance, across samples of residents in seven Latin American countries (Argentina, Ecuador, Mexico, Paraguay, Uruguay, Colombia, and El Salvador). A pattern of specific *a priori* relationships was postulated, and then its invariance across countries was examined by means of multigroup models. The second objective was to measure the levels of anxiety, depression and fear of COVID-19. According to the literature, it was expected that women would show higher levels of fear of COVID-19, anxiety and depression than men (hypothesis 1); that older people would have higher levels of fear of COVID-19, anxiety and depression (hypothesis 2); that single, separated, divorced and/or widowed people would be more likely to have symptoms of anxiety, depression and fear of COVID-19 (hypothesis 3) and that finally, fear of COVID-19 would be positively related to symptoms of depression and anxiety (hypothesis 4). See Figure 1 for the hypothesized model.

As mentioned above, the study was conducted in LAC, which is a region potentially affected by high levels of anxiety, stress, depression and fear (Arias Molina et al., 2020; Orellana and Orellana, 2020; Goularte et al., 2021), as well as with high rates of newly diagnosed cases and deaths, and where government authorities have great difficulties in meeting the health needs of the population (Acosta, 2020; Alvarez and Harris, 2020). Moreover, during the last decade, studies on the prevalence of mental disorders in LAC have focused on only a few key countries, mainly Brazil, Chile, Argentina, and Colombia (Kohn et al., 2018). Furthermore, LAC countries are underrepresented in much of the world’s leading psychiatry journals, representing less than 1% of the research produced in mental health (Patela and Sumathipala, 2001). Finally, having a model that invariantly assesses the contribution of socio-demographic factors and fear of COVID-19 on anxiety and depression in a combined sample of seven Latin American countries will allow for a better understanding, evaluation and thus improvement of interventions to address mental health problems in the population of some LAC countries during this and future pandemics. Similarly, it will not only provide an overview within each of the countries, but also comparable data to promote an exchange of information among them.

## Materials and Methods

### Design

This study used a cross-sectional and explanatory design with latent variables represented by a system of structural equations, where some variables may be observable and others are latent (Ato et al., 2013).

### Participants

This study focused on the general population residing in seven Latin American countries (Ecuador, Colombia, El Salvador, Paraguay, Mexico, Argentina, and Uruguay). The inclusion criteria were: to reside in the seven countries mentioned, to be of legal age and to have given informed consent to participate in the online study. On the other hand, the exclusion criteria were: not having Internet access and not residing in the seven Latin American countries indicated at the time of data collection. A total of 4881 individuals participated, recruited through non-probabilistic convenience sampling due to the restrictions on social interaction that were mandated in all participating countries during the time of data collection. Table 1 presents the socio-demographic characteristics of the participants in each country.

**TABLE 1:** Sample demographic characteristics by country.

	Argentina (*n* = 1719)	Colombia* (*n* = 324)	Ecuador (*n* = 790)	El Salvador (*n* = 354)	Mexico (*n* = 986)	Paraguay (*n* = 272)	Uruguay (*n* = 436)
**Sex (%)**							
Female	1351 (78.6)	241 (78.6)	512 (64.8)	250 (70.6)	701 (71.1)	217 (79.8)	333 (76.4)
Male	368 (21.4)	81 (25.2)	278 (35.2)	104 (29.4)	285 (28.9)	55 (20.2)	103 (23.6)
Age (*M* ± *SD*)	38.31 ± 15.82	33.07 ± 12.05	24.58 ± 7.76	27.79 ± 8.89	34.52 ± 11.59	36.68 ± 11.56	42.05 ± 12.98
**Relational status (%)**							
With a partner	680 (39.9)	97 (30.0%)	96 (12.2)	56 (15.9)	406 (4.4)	127 (46.9)	210 (48.3)
Single	1026 (60.1)	226 (70.0)	694 (87.8)	297 (84.1)	575 (58.6)	144 (53.1)	225 (51.7)

*Two participants did not self-identify as male or female.

### Measures

#### Socio-Demographic Information Survey

The survey was constructed specifically for this study and included questions on country of residence, age, sex, and marital status.

#### Generalized Anxiety Disorder Questionnaire (GAD-7)

This self-report measure (Spitzer et al., 2006), used in primary health care, consists of 7 items that assess the frequency of symptoms of generalized anxiety disorder (GAD) during the last 2 weeks prior to the application of the questionnaire (e.g., feeling nervous, anxious, and worried about different aspects). The items are scored on a 4-alternative Likert-type scale (0 = not at all to 3 = almost every day). The total score is obtained from the sum of the scores for each of the items and ranges from 0 to 21, where higher scores indicate the presence of more severe symptoms of generalized anxiety. Scores from 0 to 4 indicate no anxiety, 5 to 9 mild anxiety, 10 to 14 moderate anxiety, and 15 to 21 severe anxiety (Kroenke et al., 2007). In addition, a cut-off point of 10 points showed adequate values of sensitivity (86.8%) and specificity (93.4%) for the potential diagnosis of GAD. The Spanish adapted version by García-Campayo et al. (2010) was used in this study.

#### The Patient Health Questionnaire-9 (PHQ-9)

This self-report questionnaire consists of 9 items that assess the frequency of depressive symptoms during the last 2 weeks (Kroenke et al., 2001). Each item has 4 Likert-type response options (0 = not at all to 3 = almost every day). The total score is obtained from the sum of the scores for each of the items and ranges from 0 to 27, where higher scores indicate the presence of more severe depressive symptoms. From the total score, depressive symptoms are grouped into five levels of severity: 0 to 4 = minimal, 5 to 9 = mild, 10 to 14 = moderate, 15 to 19 = moderately severe, and 20 to 27 = severe. A cutoff point ≥ 8 (sensitivity 88.20%, specificity 86.60%, and PPV 90.91%) is considered optimal for the diagnosis of a major depressive episode (MDE). The Spanish adapted version by Urtasun et al. (2019) was used in this study.

#### Fear of COVID-19 Scale (FCV-19S)

This self-report scale consists of 7 items that assess fear of COVID-19. Each item has 5 Likert-type response alternatives, ranging from 1 = strongly disagree to 5 = strongly agree. Higher scores indicate higher levels of fear of COVID-19 (Ahorsu et al., 2020). The total score is calculated from the sum of the scores for each item and ranges from 7 to 35, where a higher score indicates a higher fear of COVID-19. In this study, the version adapted and cross-culturally validated in different Latin American countries was used (Caycho-Rodríguez et al., 2021b). A meta-analysis study, which evaluated 42 studies from various countries, indicated that Cronbach’s alpha coefficients ranged from 0.85 to 0.90 (Blázquez-Rincón et al., 2021). All the questions of the measures used are shown in the Appendix.

### Procedure

An online questionnaire was designed on the Google Forms platform, which was disseminated via email and social networks, such as Facebook and Instagram. Each link detailed the objective of the study. The confidentiality of the participants was guaranteed and they gave their informed consent before answering the survey questions.

Data were collected between June 12 and September 14. During this time period, each country experienced different phases of the COVID-19 pandemic. In Ecuador, data collection was conducted between June 14 and September 13, when the country was in a period known as risk zones, based on the number of diagnosed cases occurring in each region. During this period, a decrease in the infection curve was observed, reaching 2,053 confirmed cases on September 13. In Argentina, data were collected between June 12 and September 13, during the change from phase IV to phase V, which was characterized by the reopening of economic and commercial activities. During this period, the infection curve showed a gradual and steady increase, with a peak of 12,259 cases per day on September 9. For this reason, the Argentine government tightened restrictive measures, moving back to phases I and II in some provinces of the country. In Uruguay, data collection was carried out between June 16 and September 13, when the country was in the process of reopening its activities. During this period, no restrictions or phase reversals were observed and the peak of infection was on July 21 with a total of 29 confirmed cases. In Paraguay, data were collected between July 2 and September 11, a period in which the country was at the end of phase III and the beginning of phase IV of intelligent isolation. During this period, a gradual increase in the infection curve was observed, reaching a peak of 1,217 confirmed cases on September 5, which generated a regression to phase III in several regions of the country. In Colombia, the collection process took place between June 14 and September 3, when the country was in mandatory isolation, with some opening of economic activities and setbacks. During this period, there was an increase in the number of confirmed cases, reaching 13,056 cases on August 19. From September 1, the country was fully opened and on the last day of the collection period (September 3), 8,024 cases were reported. In Mexico, collection took place between June 14 and September 14, which corresponds to the beginning of the so-called “New Normal.” During this period, the peak of infection occurred on August 01, with 9,556 infections, with a subsequent decrease in the infection curve to an average of 3,500 cases per day. Finally, in El Salvador, data collection took place between August 7 and September 9, a period characterized by a decrease in the number of cases. Thus, in August, a set of protocols for the proper use of public spaces were published. The highest number of cases was observed on August 14 (449 confirmed cases).

### Statistical Analyses

First, descriptive statistics were calculated for all the study variables. Specifically, means and standard deviations were calculated for quantitative variables and frequencies and percentages for categorical variables. These calculations were performed with SPSS 23. A completely *a priori* Robust Structural Equation Model (SEM) was then tested in the overall sample. This model is presented in Figure 1. WLSMV (Weighted Least Squares Mean and Variance corrected) was the chosen method of estimation given the lack of multivariate normality and the ordinal nature of the items included in the model (Hancock and Mueller, 2013). Model fit was assessed with different indexes and statistics from different families (Tanaka, 1993): (a) the chi-square test of model fit; (b) the Comparative Fit Index (CFI); (c) the Standardized Root Mean Residual (SRMR); and (d) the Root Mean Square Error of Approximation (RMSEA) with a 90% confidence interval. We used the following criteria for declaring good model fit: CFI above 0.90 (better fit above 0.95), and RMSEA and SRMR below 0.08 (Marsh et al., 2004). Given that we had samples from 7 different Central and South American countries, data were further analyzed with a multigroup Structural Equation Model by country. In this multigroup routine, three models were tested, with each model in the routine adding constraints across countries (van de Schoot et al., 2012). First, a configural model was tested in which the model was estimated in all countries at the same time but separately. Therefore, there are no constrains across countries. This model gives us the baseline fit. Then, all factor loadings of the items for anxiety, depression, and fear of COVID-19 were set as equal across countries. This is a pre-requisite for testing moderation effects across countries. Finally, a third structural model was tested in which all effects among observed and latent variables were constrained to be equal across countries. The models in this sequence are nested and may be compared with a formal statistical test or chi-square differences, with a modeling strategy or CFI differences (Little, 1997). No chi-square differences or CFI differences of less than 0.01 support the more parsimonious (more constrained) model (Cheung and Rensvold, 2009). All structural equation models were estimated in Mplus 8.5 (Muthén and Muthén, 1998-2017). Cronbach’s alpha was used to evaluate the reliability of the questionnaires used in the survey.

### Ethics

The study was conducted in accordance with the principles of the Declaration of Helsinki. Additionally, the study protocol was approved by the Ethics Committee at the Universidad Privada del Norte (protocol number: 20213002-UPN-DNID).

## Results

First, Table 2 shows the mean, standard deviation, range of scores and reliability estimates. All instruments have high levels of reliability in each of the countries. Second, 31.40% of the total participants did not present symptoms of generalized anxiety, 43% presented mild anxiety, 17.20% moderate anxiety and 8.30% severe anxiety. Regarding depressive symptoms, 41.30% presented minimal symptoms of depression, 31.20% mild depression, 15.20% moderate depression, 7.60% moderately severe depression and 3.90% severe depression. Using a cut-off score of 10 for the GAD-7, we found that 1,245 participants (25.50%) presented symptoms of GAD. Furthermore, using a cutoff score ≥ 8 for the PHQ-9, we observed that 1,825 (37.39%) presented a MDE. The mean FCV-19S score for the total number of participants was 15.54 (*SD* = 6.64). Table 3 presents the levels of generalized anxiety and depression for each of the participating countries.

**TABLE 2:** Descriptive statistics of the GAD-7, PHQ-9, and FCV-19S.

	Argentina	Colombia	Ecuador	El Salvador	Mexico	Paraguay	Uruguay
**GAD-7**							
*M*	7.59	6.23	7.18	7.27	6.71	8.02	5.49
*SD*	4.73	4.37	4.10	4.96	4.54	4.94	4.03
Range	0–21	0–21	0–21	0–21	0–21	0–21	0–21
α	0.85	0.85	0.84	0.88	0.86	0.86	0.86
**PHQ-9**							
*M*	7.57	6.63	7.01	6.92	6.45	7.20	5.09
*SD*	5.74	5.96	5.51	5.90	5.46	6.03	5.04
Range	0–27	0–26	0–27	0–27	0–27	0–27	0–27
α	0.88	0.91	0.89	0.89	0.89	0.89	0.90
**Fear of COVID-19**							
*M*	13.63	15.90	17.97	17.76	17.17	16.22	12.48
*SD*	5.64	6.51	6.94	7.59	6.86	6.05	6.64
Range	7–35	7–35	7–35	7–35	7–35	7–35	7–35
α	0.83	0.87	0.88	0.89	0.87	0.82	0.85

*M, mean; SD, standard deviation; α, Cronbach’s alpha.*

**TABLE 3:** Levels of generalized anxiety and depression.

	Argentina (*n* = 1719)	Colombia (*n* = 324)	Ecuador (*n* = 790)	El Salvador (*n* = 354)	Mexico (*n* = 986)	Paraguay (*n* = 272)	Uruguay (*n* = 436)
**Generalized anxiety (%)**							
No anxiety (0–4 points)	487 (28.3)	126 (38.9)	214 (27.1)	113 (31.9)	337 (34.2)	65 (23.9)	193 (44.3)
Mild anxiety (5–9 points)	723 (42.1)	142 (43.8)	375 (47.5)	132 (37.3)	413 (41.9)	120 (44.1)	196 (45.0)
Moderate anxiety (10–14 points)	331 (19.3)	36 (11.1)	144 (18.2)	76 (21.5)	173 (17.5)	53 (19.5)	28 (6.4)
Severe anxiety (15–21 points)	178 (10.4)	20 (6.2)	57 (7.1)	33 (9.3)	63 (6.4)	34 (12.5)	19 (4.4)
TAG (≥10 points)	509 (29.6)	56 (17.3)	201 (25.4)	109 (30.8)	236 (23.9)	87 (32.0)	47 (10.8)
**Depression (%)**							
Minimum (0–4 points)	615 (35.8)	147 (45.4)	303 (38.4)	156 (44.1)	437 (44.3)	115 (42.3)	243 (55.7)
Mild (5–9 points)	569 (33.1)	101 (31.2)	282 (35.7)	95 (26.8)	310 (31.4)	73 (26.8)	137 (31.4)
Moderate (10–14 points)	308 (17.9)	39 (12.0)	120 (15.2)	60 (16.9)	140 (14.2)	47 (17.3)	27 (6.2)
Moderately severe (15–19 points)	146 (8.5)	21 (6.5)	57 (7.2)	31 (8.8)	70 (7.1)	26 (9.6)	18 (4.1)
Severe (20–27 points)	81 (4.7)	16 (4.9)	28 (3.5)	12 (3.4)	29 (2.9)	11 (4.0)	11 (2.5)
EDM (≥8 points)	724 (42.12)	106 (32.72)	315 (39.87)	135 (38.14)	339 (34.38)	106 (38.97)	100 (22.94)

Second, a completely *a priori* SEM was tested in the overall sample. This model has two latent response variables, anxiety and depression. They are predicted by a latent variable of fear of COVID-19, and three socio-demographic variables: age, sex and living or not with a partner. This *a priori* SEM fit the data extremely well: c^2^(287) = 5936.96, *p* < 0.001; RMSEA = 0.064 [0.062, 0.065]; CFI = 0.947; and SRMR = 0.050.

**FIGURE 2:**
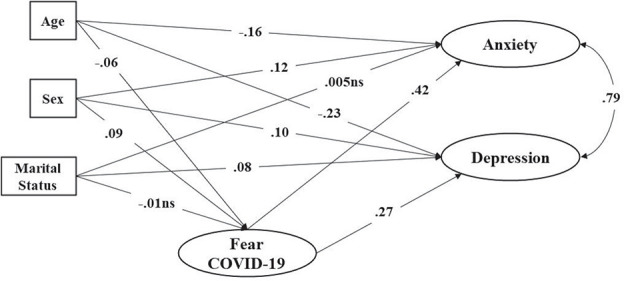
Structural Equation Model of fear of COVID-19 predicting anxiety and depression. For the sake of clarity factor loadings and errors not shown; all estimates *p* < 0.01 unless stated as ns (non-significant).

The parameter estimates for this SEM are presented in Figure 2, with the exception of factor loadings which are shown in Table 4. Fear of Covid-19 significantly and positively predicted both anxiety and depression. The impact is larger on anxiety than on depression. Regarding the effects of the socio-demographics, their effects are, in general, low. As people age, they have less fear of COVID, anxiety and depression. Women had, on average, more fear of COVID and depression, but the same level of anxiety as men. Living with a partner was not significantly related with anxiety and fear of COVID, but was significantly related with being depressed.

**TABLE 4:** Standardized factor loadings for all the latent variables.

Item	Fear of COVID	Anxiety	Depression
1	0.775	0.814	0.765
2	0.667	0.612	0.894
3	0.754	0.786	0.737
4	0.778	0.818	0.818
5	0.820	0.757	0.712
6	0.845	0.760	0.807
7	0.879	0.736	0.749
8			0.765
9			0.689

Once the SEM was estimated in the total sample, a multigroup sequence of models, as explained in the statistical analyses section, was tested. Goodness-of-fit indexes are presented in Table 5. According to these indices, especially the chi-square and CFI differences, it is clear that there is no evidence of cross-country moderation effects. In other words, the results found in the total sample remain the same across the Central and South American countries analyzed.

**TABLE 5:** Model fit indexes.

Models	χ^2^	df	*p*	Δχ^2^	Δdf	*p*	RMSEA [CI 90%]	SRMR	CFI	ΔCFI
Configural	7085.353	2309	<0.001	–	–	–	0.055 [0.053, 0.056]	0.058	0.954	–
Equal loadings	7003.517	2429	<0.001	218.8	120	<0.001	0.052 [0.051, 0.054]	0.057	0.956	0.002
Equal structural effects	6171.319	2501	<0.001	111.239	72	=0.002	0.046 [0.045, 0.047]	0.063	0.965	0.009

*The chi-square difference tests and CFI differences are comparing with the nearest less constrained model.*

## Discussion

This study proposes, and tests, a model relating socio-demographic variables, fear of COVID-19, anxiety symptoms, and depression in the general population of seven Latin American countries during the COVID-19 pandemic. Multigroup analyses showed that the proposed model fit the data in all countries. Therefore, the relationships among the variables show no differences among the seven countries. This is important in cross-cultural research, as comparisons between different cultures and/or countries would not be valid if measurement invariance is not met (Milfont and Fischer, 2010).

In the present study, 25.5% of the participants from the seven Latin American countries presented moderate and severe levels of anxiety and 26.7% presented moderate and severe levels of depression. These results are below those reported in previous research. For example, a systematic and meta-analytic review indicated a prevalence of anxiety at 31.90% (95% confidence interval: 27.50–36.70) and 33.70% for depression (95% confidence interval: 27.50–40.60) (Salari et al., 2020). Another systematic review, which evaluated 19 studies with a total of 93,569 participants, reported relatively high rates of anxiety symptoms (6.33–50.90%) and depression (14.60–48.30%). Similarly, a study conducted in a combined population of 113,285 people indicated that the prevalence of depressive and anxiety symptoms was 20 and 35%, respectively (Lakhan et al., 2020). In the case of fear of COVID-19, the mean score of the total sample (*M* = 15.54, *SD* = 6.64) was lower than reported in other contexts such as, for example, India (*M* = 18.00, *SD* = 5.68; Doshi et al., 2020) and an Amharic-speaking population (*M* = 20.79, *SD* = 5.78 to *M* = 21.65, *SD* = 5.58; Elemo et al., 2020). Likewise, these findings are also consistent with previous research that reported how exposure to other public health problems such as the Ebola outbreak (Shultz et al., 2015) and SARS (Mak et al., 2009) can generate mental health problems. The lower levels in the Latin American context can be explained, in part, by the ample information about the virus in this part of the world. LAC was the last region to have cases diagnosed with the disease, so such knowledge about the pandemic could explain the lower levels of anxiety, depression and fear. However, it is to be expected that reported levels of depression, anxiety and fear will increase as confinement and isolation expand, so it would be useful to analyze this trend over time (Brooks et al., 2020). Even so, the findings suggest that the COVID-19 pandemic has affected the mental health of people in the countries assessed. In this regard, high levels of anxiety and depression during the pandemic may be problematic due to their strong association with alterations in physical activity, sleep, as well as increased tobacco and alcohol consumption (Stanton et al., 2020). An analysis by country indicates that Uruguay has the lowest percentages of people with moderate and severe anxiety (10.80%) and moderate and severe levels of depression (12.80%), as well as the lowest average fear score for COVID-19 (*M* = 12.48). One explanation for this could be the successful management of the pandemic by the Uruguayan government. In this sense, having a relatively small population of approximately 3.5 million inhabitants has facilitated the control of COVID-19 transmission, making Uruguay one of the countries with the fewest diagnosed cases and deaths from COVID-19 (Taylor, 2020; Caycho-Rodríguez et al., 2021b). Similarly, cultural differences and available information on the consequences of COVID-19 may also explain differences in the prevalence of symptoms of generalized anxiety, depression and fear (Bäuerle et al., 2020).

Regarding the impact of socio-demographic variables, it was found that older people have fewer symptoms of anxiety, depression, and fear of COVID-19. This finding is consistent with studies suggesting that older ages are associated with less negative emotional responses to the COVID-19 pandemic (Salari et al., 2020; Bruine de Bruin, 2021). Some suggest that younger people are more concerned about future consequences and economic problems caused by the pandemic, as they are profoundly affected by layoffs and business closures (Ahmed et al., 2020; Huang and Zhao, 2020). In addition, higher levels of anxiety and stress among younger people would also be related to greater access to information about the pandemic through social networks (Scholten et al., 2020). In contrast, despite the negative consequences of the COVID-19 pandemic, older people seem to have regulated their emotions by focusing them on the positive and engaging in stress-reducing activities (Neubauer et al., 2019). However, it should be considered that while optimism allows for better regulation of emotions in the short term, it may sometimes fail to prepare people to cope with future negative outcomes (Shepperd et al., 2015).

As expected, gender had an impact on the mental health of the participants, where women presented more symptoms of depression and fear of COVID-19. This is consistent with previous studies that have shown a higher frequency of depressive symptoms and fear of COVID-19 in women (Broche-Pérez et al., 2020; Özdin and Bayrak Özdin, 2020; Rossi et al., 2020; Ausín et al., 2021). This seems to indicate that women might be suffering a greater burden of care both inside and outside the home during the pandemic (McLaren et al., 2020). In addition, the results could also be associated with greater reactivity of women in neural networks related to fear responses (Liu N. et al., 2020). Similarly, there are hormonal differences that may explain the results (de Arrieta and Arenaza, 2019). Other studies suggest that while women are more adaptable to environmental stressors, they tend to be physically weaker and get sick more often than men (Overfield, 2018). The presence of illness increases concerns about possible COVID-19 contagion and increases psychological burden, both in individuals and in the general population (Musche et al., 2020). Thus, getting sick more often may have increased the perception of risk and levels of fear related to COVID-19 in women compared to men (Bakioğlu et al., 2020). Indeed, gender differences with respect to risk perception are expressed in behavioral differences between men and women (Rodriguez-Besteiro et al., 2021). On the other hand, men may avoid expressing their fears due to gender roles, which emphasize the strength and bravery of the male gender (Bakioğlu et al., 2020). These findings may provide information for health policy formulation in the countries involved. Thus, since depression is a priority mental health problem, it is important to understand which subgroups have a greater need for services (Salk et al., 2017). Therefore, universal screening for depressive symptoms in primary care settings with a strong emphasis on the female group is needed (O’Connor et al., 2009).

Women had the same levels of anxiety symptoms as men. This is contrary to previous studies reporting three times higher levels of anxiety in women than in men during the pandemic (Liu N. et al., 2020; Wang et al., 2020). One possible explanation for this could be that, as a result of confinement, household responsibilities (childcare, cooking, cleaning, etc.) are shared between men and women. The disinclination of men in the countries included in this study to perform domestic activities can generate difficulties in the management of personal, professional and family life, which can make them just as or even more anxious than women (Verma and Mishra, 2020). Housework can be considered as routine and boring, so it can have negative effects on well-being and health, both for women and men (Arbide et al., 2009), although in the latter, the lack of habit in performing this type of activities may seem to generate a greater impact. However, these results should be analyzed on the basis of domestic inequalities, which are particularly marked in countries with low levels of gender equality and female empowerment (Fuwa, 2004; United Nations, 2020). In this sense, as a future line of research, future studies should analyze the influence that gender roles and stereotypes have on the presence of anxiety symptoms related to COVID-19. Finally, living with a partner was not significantly related to anxiety and fear of COVID-19, but was significantly related to depression. This finding is in line with what has been reported in previous literature, where significantly higher odds of having depressive symptoms were observed in the married or partnered group, which could be explained because they not only care about themselves, but there is also a greater sense of responsibility and concern for the well-being of the partner (Doshi et al., 2020; Pérez et al., 2020). However, researchers suggest that vulnerability to the development of depression is not only related to marital status, but may be modified by gender and age; therefore, it is recommended to evaluate models to quantify these modifications (Bulloch et al., 2017).

On the other hand, fear is an emotion that affects physical responses, cognitive abilities, and mood (Bakioğlu et al., 2020). This could explain the findings of the present study which indicated that fear of COVID-19 increases the levels of anxiety and depression in the general population of the Latin American countries involved. This relationship is not surprising and is consistent with previous studies (Ahorsu et al., 2020; Alyami et al., 2020; Bitan et al., 2020; Mertens et al., 2020; Shigemura et al., 2020). This suggests that people with fear of COVID-19, which has greater infectiousness and more negative consequences than other viral respiratory diseases, have higher levels of anxiety and depression (Bakioğlu et al., 2020). In short, a negative emotion, such as fear of COVID-19, triggers others that may further aggravate people’s mental health (Satici et al., 2020a). These findings can be explained by uncertainty, the belief that the pandemic should not be controlled, the severity of the disease, fear of becoming infected, information deficits, social isolation, and economic problems generated by the pandemic that influence the presence of fear, anxiety, and depression among the general population (Shigemura et al., 2020; Zandifar and Badrfam, 2020; Sakib et al., 2021). Furthermore, people with higher levels of fear may not be able to think rationally to mitigate the presence of COVID-19 anxiety symptoms (Green et al., 2021). Having evidence that fear of COVID-19 can predict negative psychological reactions, such as anxiety and depression, is important because these psychological reactions decrease well-being and life satisfaction, more so in circumstances such as the current pandemic (Alyami et al., 2020). Furthermore, depression and anxiety play a mediating role in the relationship between fear of COVID-19 and life satisfaction (Satici et al., 2020b). Likewise, this finding would support the development of strategies to minimize the psychological impact that fear, depression and anxiety could cause in the Latin American countries studied (de Medeiros et al., 2021). Mental health problems during public health emergencies related to infectious diseases, such as COVID-19, could be related to a misinterpretation of harmless bodily sensations or changes associated with health as symptoms of the disease, causing people to become unduly distressed (Taylor, 2019).

This study has some limitations. First, the countries were not selected systematically in the study. The inclusion of countries was the result of a negotiation of co-author interest in participating in the study and their capacity to meet the requirements of the proposed design. Second, the design was cross-sectional in nature and it would be interesting to conduct a study with a longitudinal design to track variations in the relationships between depression, anxiety, and fear of COVID-19 in participants from all countries during later stages of the pandemic. Third, data were collected mostly from urban settings in each country, so results may vary in rural settings or settings with lower population density and higher risk of infection. Fourth, participants from Ecuador, Colombia, El Salvador, Paraguay, Mexico, and Argentina showed higher levels of generalized anxiety, depression and fear of COVID-19; however, there was no information on the pre-existence of mental illness in the respondents. Elevated levels of stress and anxiety in participants could have existed before this study was conducted due to information through the media, more so because the pandemic has affected several American and European countries (Salari et al., 2020). For example, before the pandemic, people from Ibero/Latin regions, showed a prevalence of general anxiety disorders of 6.20% (Remes et al., 2016). Additionally, it is possible that someone who has experienced anxiety or depression prior to the pandemic is predisposed to be fearful or worried about the impact of COVID-19. In this regard, future studies could address this and investigate the connection between anxiety and depression as predictors of fear of COVID-19. Fifth, preparedness to face the pandemic has varied among the different countries in Latin America, making them vulnerable to the disease due to the limited resources of their health care systems, the late responses of governments and the high rates of poverty and inequality (Burki, 2020; Pablos-Méndez et al., 2020). All these factors would affect the transmission and impact of COVID-19 in Latin America, which also has implications for the mental health of the population. Therefore, the different infection and death curves for COVID-19 in the participating countries during the data collection time period could have led to an over- or underestimation of the presence of the mental health symptoms evaluated. Sixth, the non-probabilistic nature of the sampling did not allow for a fully representative sample of the population of each of the countries. In addition, there was a risk of sampling bias since it was not possible to survey people without internet access in all the countries involved. On the other hand, although the participants in each country were recruited in the same way, the distribution of demographic variables was different. These demographic differences could be corrected by using appropriate sampling (Pierce et al., 2020). A seventh limitation is that the reliability of diagnoses made with the GAD-7, PHQ-9, and FCV-19S may vary between countries and, therefore, the accuracy of the diagnoses may vary. An eighth limitation lies in the use of self-report measures to assess levels of generalized anxiety, depression, and fear of COVID-19, which are not always related to objective assessments by mental health professionals. However, as anxiety, depression, and fear are based on personal emotions, self-assessment measures have been important during the COVID-19 pandemic as information-gathering techniques (Wang et al., 2020). Another limitation includes the possible systematic effect of the data collection method. Although the effect of mode of administration was not assessed, it may potentially interact with cultural effects in each country. Thus, future studies using different forms of survey administration (pencil and paper and online) would allow for separating the effect of administration reliably (Żemojtel-Piotrowska et al., 2018). Finally, other variables that could be useful to explain the model such as intolerance to uncertainty (Bakioğlu et al., 2020), educational level (Chen et al., 2020) or economic income level (Rudenstine et al., 2021) were not included.

Despite these limitations, the strengths of this study include the use of a large number of participants, the use of psychometric instruments that have demonstrated cross-cultural validity for measuring generalized anxiety (Plummer et al., 2016), depression (Manea et al., 2012; Blackwell and McDermott, 2014) and fear of COVID-19 (Caycho-Rodríguez et al., 2021b), as well as the use of statistical methods that consider all variables within the same analysis. In addition, the study addresses the relationships between socio-demographic and psychological variables based on previous research and provides important information for mental health professionals, public policy makers and researchers (Holmes et al., 2020).

## Conclusion

This study of thousands of participants from seven Latin American countries suggests that fear of COVID-19 significantly and positively predicts both anxiety and depression, while the effects of socio-demographic variables are low. In addition, it was observed that about a quarter of the participants presented symptoms of GAD and a MDE. This suggests the need for the implementation of preventive actions in the general population of these countries, with the aim of reducing the prevalence of depressive, anxious and fearful symptoms related to COVID-19. In this sense, it is important to provide care for people who have moderate or severe mental health problems (depression, anxiety, or fear of COVID-19), as well as to develop strategies aimed at people with mild levels, and thus prevent them from progressing to more severe stages. Similarly, it is important to implement national policies and epidemiological surveillance strategies for fear of COVID-19, depressive and anxious symptoms.

Thus, we recommend the use of technological tools such as applications or short online self-assessment systems to collect information on emotional problems (anxiety, stress, or depression) of the general population. For example, at the Latin American level, Integrative Community Therapy (ICT) has been developed as an online psychosocial intervention within the public health system with the aim of strengthening and building support networks, minimizing stigma and prejudice toward people affected by COVID-19 and giving hope to those in social confinement (de Paula Barreto et al., 2020). In Mexico, an intervention based on positive psychology is being carried out through a web platform to reduce anxiety and depression symptoms and increase positive symptoms (Dominguez-Rodriguez et al., 2020). Working on the basis of positive emotions fosters the development of long-term personal coping resources to promote self-improvement, greater well-being and post-epidemic growth (Fredrickson et al., 2003). Additionally, it would be important to test the efficacy of interventions developed in other contexts such as China, where an online psychological-behavioral intervention program was developed, including psychological support and breathing exercises, which showed beneficial effects on the mental health of patients with COVID-19 (Kong et al., 2020). In addition, psychological counseling services and mental health education information can be shared online with programs such as WeChat, Weibo, and TikTok, which have already been widely used (Liu S. et al., 2020). These types of strategies have proven useful in addressing mental health needs and identifying people with severe emotional problems in different countries during the COVID-19 pandemic (Torous and Keshavan, 2020; Wang et al., 2021). Even so, future studies are required to replicate these findings in samples from other Latin American and/or European countries, with the aim of identifying those factors that explain the effect of country of residence on some mental health indicators and to improve the understanding of variations in mental health, both at the country and individual level. Online interventions should be systematically evaluated according to established criteria for digital mental health studies, which will inform the quality of these interventions. Finally, consideration should be given to inequalities and potential drawbacks, such as limited access to technologies, educational inequities, or cultural peculiarities, which may limit access to and use of digital mental health intervention platforms.

## Data Availability Statement

The original contributions presented in the study are included in the article/Supplementary Material, further inquiries can be directed to the corresponding author.

## Ethics Statement

The studies involving human participants were reviewed and approved by Universidad Privada del Norte. The patients/participants provided their written informed consent to participate in this study.

## Author Contributions

TC-R, MCe, MG, PM, IB, and MCa provided initial conception, organization, and main writing of the text. JT, LV, and CC-L analyzed the data and prepared all figures and tables. CA, CL-C, RM-H, EC-A, ML-R, PC, YQ, ER, ML, OR, AF, MW, and CV were involved in data collection for their respective countries and acted as consultants and contributors to research design, data analysis, and text writing. They read and approved the draft. All authors contributed to the article and approved the submitted version.

## Conflict of Interest

OR was employed by company Sensorium Corp. The remaining authors declare that the research was conducted in the absence of any commercial or financial relationships that could be construed as a potential conflict of interest.

## Publisher’s Note

All claims expressed in this article are solely those of the authors and do not necessarily represent those of their affiliated organizations, or those of the publisher, the editors and the reviewers. Any product that may be evaluated in this article, or claim that may be made by its manufacturer, is not guaranteed or endorsed by the publisher.

## Appendix

### Socio-Demographic Information Survey

1. Country of residence (Argentina, Colombia, Ecuador, El Salvador, México, Paraguay, and Uruguay).
2. Age.
3. Sex (Female and male).
4. Relational status (With a partner and Single).

### Generalized Anxiety Disorder Questionnaire

1. Feeling nervous, anxious or on edge?
2. Not being able to stop or control worrying?
3. Worrying too much about different things?
4. Trouble relaxing?
5. Being so restless that it is hard to sit still?
6. Becoming easily annoyed or irritable?
7. Feeling afraid as if something awful might happen?

### The Patient Health Questionnaire-9

1. Little interest or pleasure in doing things?
2. Feeling down, depressed, or hopeless?
3. Trouble falling or staying asleep, or sleeping too much?
4. Feeling tired or having little energy?
5. Poor appetite or overeating?
6. Feeling bad about yourself — or that you are a failure or have let yourself or your family down?
7. Trouble concentrating on things, such as reading the newspaper or watching television?
8. Moving or speaking so slowly that other people could have noticed? Or so fidgety or restless that you have been moving a lot more than usual?
9. Thoughts that you would be better off dead, or thoughts of hurting yourself in some way?

### Fear of COVID-19 Scale

1. I am most afraid of coronavirus-19.
2. It makes me uncomfortable to think about coronavirus-19.
3. My hands become clammy when I think about coronavirus-19.
4. I am afraid of losing my life because of coronavirus-19.
5. When watching news and stories about coronavirus-19 on social media, I become nervous or anxious.
6. I cannot sleep because I’m worrying about getting coronavirus-19.
7. My heart races or palpitates when I think about getting coronavirus-19.
